# Metabarcoding options to study eukaryotic endoparasites of birds

**DOI:** 10.1002/ece3.7748

**Published:** 2021-07-02

**Authors:** Vincent Bourret, Rafael Gutiérrez López, Martim Melo, Claire Loiseau

**Affiliations:** ^1^ CIBIO‐InBIO Centro de Investigação em Biodiversidade e Recursos Genéticos Universidade do Porto Vairão Portugal; ^2^ MHNC‐UP – Natural History and Science Museum of the University of Porto Porto Portugal; ^3^ FitzPatrick Institute of African Ornithology University of Cape Town Cape Town South Africa; ^4^ CEFE Université de Montpellier CNRS Montpellier France

**Keywords:** birds, metabarcoding, parasites, primers

## Abstract

There is growing interest in the study of avian endoparasite communities, and metabarcoding is a promising approach to complement more conventional or targeted methods. In the case of eukaryotic endoparasites, phylogenetic diversity is extreme, with parasites from 4 kingdoms and 11 phyla documented in birds. We addressed this challenge by comparing different primer sets across 16 samples from 5 bird species. Samples consisted of blood, feces, and controlled mixes with known proportions of bird and nematode DNA. Illumina sequencing revealed that a 28S primer set used in combination with a custom blocking primer allowed detection of various plasmodiid parasites and filarioid nematodes in the blood, coccidia in the feces, as well as two potentially pathogenic fungal groups. When tested on the controlled DNA mixes, these primers also increased the proportion of nematode DNA by over an order of magnitude. An 18S primer set, originally designed to exclude metazoan sequences, was the most effective at reducing the relative number of avian DNA sequences and was the only one to detect *Trypanosoma* in the blood. Expectedly, however, it did not allow nematode detection and also failed to detect avian malaria parasites. This study shows that a 28S set including a blocking primer allows detection of several major and very diverse bird parasite clades, while reliable amplification of all major parasite groups may require a combination of markers. It helps clarify options for bird parasite metabarcoding, according to priorities in terms of the endoparasite clades and the ecological questions researchers wish to focus on.

## Graphical abstract

Parasites of birds are extremely diverse, spanning no less than four different kingdoms (Chromista, Protozoa, Metazoa and Fungi). Our understanding of these communities and their fascinating interactions could benefit from metabarcoding approaches. This report provides a side‐by‐side comparison of different metabarcoding options for a broad description of avian eukaryotic endoparasite communities.

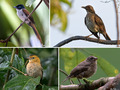

## 1 INTRODUCTION

Parasites can co‐exist within their host in the form of rich communities or assemblages that entertain different ecological interactions, from facilitation to competition and exclusion (Clark et al., 2016; Pedersen & Fenton, 2007). There can be multiple incentives to describe these assemblages, from the point of view of parasite community ecology or host health and fitness consequences of multiple infections (Bordes & Morand, 2011; Marzal et al., 2008; de Roode et al., 2005). In birds, there is growing interest in the study of eukaryotic endoparasite communities for poultry farming (Hauck, 2017), conservation biology (Carlson et al., 2011; Riper et al., 1986), ecological history (Cornuault et al., 2012; Hellgren et al., 2011), descriptive epidemiology (Santiago‐Alarcon et al., 2008), or biodiversity and biogeography of avian parasites (Enslow et al., 2020; Fecchio et al., 2019). To date, most studies have focused on selected parasite groups, potentially missing interactions beyond these groups for lack of a practical approach to assess broader parasite diversity. Such interactions between phylogenetically distant parasite groups are very likely in birds; for instance, positive and negative correlations between avian malaria apicomplexans and microfilaria blood parasites have been observed using conventional methods (Clark et al., 2016). Likewise, interactions between microparasites and helminths from different tissues (bloodstream and digestive tract) demonstrated in other hosts may also be consequential (Graham, 2008; Knowles, 2011).

Molecular approaches have greatly facilitated research across a broad diversity of parasitic taxa and may help unravel the huge and largely undescribed diversity of bird parasites (Carlson et al., 2020). Metabarcoding in particular has become a standard approach to describe biological communities in a variety of contexts (Deiner et al., 2017; Pollock et al., 2018; Xu, 2016) and holds potential for surveying parasite communities. Metabarcoding involves amplifying a small (up to a few hundred bases long) genetic marker (“the barcode”) using a pair of primers that will anneal in all target taxa. Next‐generation sequencing technology is then used to sequence the diversity of barcode variants obtained. These sequences are searched against a reference database (e.g., the Silva or the BOLD databases) to identify the taxa amplified from the sample whose sequence is also present in the database. The primer pair is a defining feature of the barcode and will determine which taxa are amplified and thus detected as part of the sampled community. Finding and using adequate primers is therefore key to the success of any metabarcoding enterprise.

Exhaustive description of parasite communities on a large scale may be difficult with traditional techniques such as microscopy, and there can be important benefits of applying a metabarcoding approach to parasite identification. For instance, it enables detection of all parasite life stages; it makes high throughput processing of samples possible; and DNA sequences are easy to share between laboratories (Aivelo, 2015; Aivelo & Medlar, 2018). As such, metabarcoding is being used extensively to study bacterial communities, with the 16S rRNA gene being the barcode of choice across many host taxonomic groups, including birds (Ganz et al., 2017; Leclaire et al., 2019). Studies using metabarcoding for eukaryotic endoparasite characterization are much less common however. Most have been conducted in mammals, where they aimed at identifying fecal parasites (Aivelo & Medlar, 2018; Avramenko et al., 2018; Gogarten et al., 2020). In birds, where blood parasites such as apicomplexans also have a major importance, parasite metabarcoding studies investigating both fecal and blood samples are missing.

The phylogenetic diversity of bird eukaryotic endoparasites is extreme and spans no less than four different kingdoms. It includes unicellular parasites from both the Chromista and Protozoa kingdoms, fungi, and metazoans (birds, together with bony fish, have been predicted to harbor the largest number of undescribed helminth species; Carlson et al., 2020). The major issue preventing bird parasite metabarcoding investigations is the lack of a practical primer set to capture this astounding diversity, while not amplifying excessive amounts of unwanted targets such as host DNA. This issue stems from the fact that some major parasites groups (e.g., helminths) are phylogenetically closer to the avian host than they are from other parasites of interest (e.g., protozoans) and is especially acute with blood samples, where red blood cells are nucleated. Therefore, the aim of this study was to test different candidate metabarcoding primer sets for their ability to recover the broadest complement of parasites from major clades, in both blood and fecal samples, despite large quantities of nontarget (host) DNA being present.

From the literature, we selected five different primers sets that have potential to detect diverse bird‐infecting parasite clades. Some are targeted at the 18S rDNA, either for all eukaryotes or for single‐celled eukaryotes only, while others are targeted at the 28S rDNA region and were complemented with custom blocking primers to reduce amplification of host sequences. We then carried out direct laboratory comparisons using a set of samples including bird blood and fecal DNA extracts, as well as artificial mixes consisting of bird blood DNA spiked in with different proportions of nematode DNA.

## 2 MATERIALS AND METHODS

### 2.1 Samples

Sixteen samples were used for this study, consisting of:

‐ Eight passerine bird blood DNA samples, known to be positive for hemosporidian parasites, based on the cytochrome *b* nested PCR described in Reis et al. (2021).
‐ Six bird fecal DNA samples.
‐ Two artificial mixes consisting of hemosporidian‐negative bird blood DNA spiked with different proportions of nematode DNA (1:100 or 1:1,000 by mass).

**FIGURE 1:**
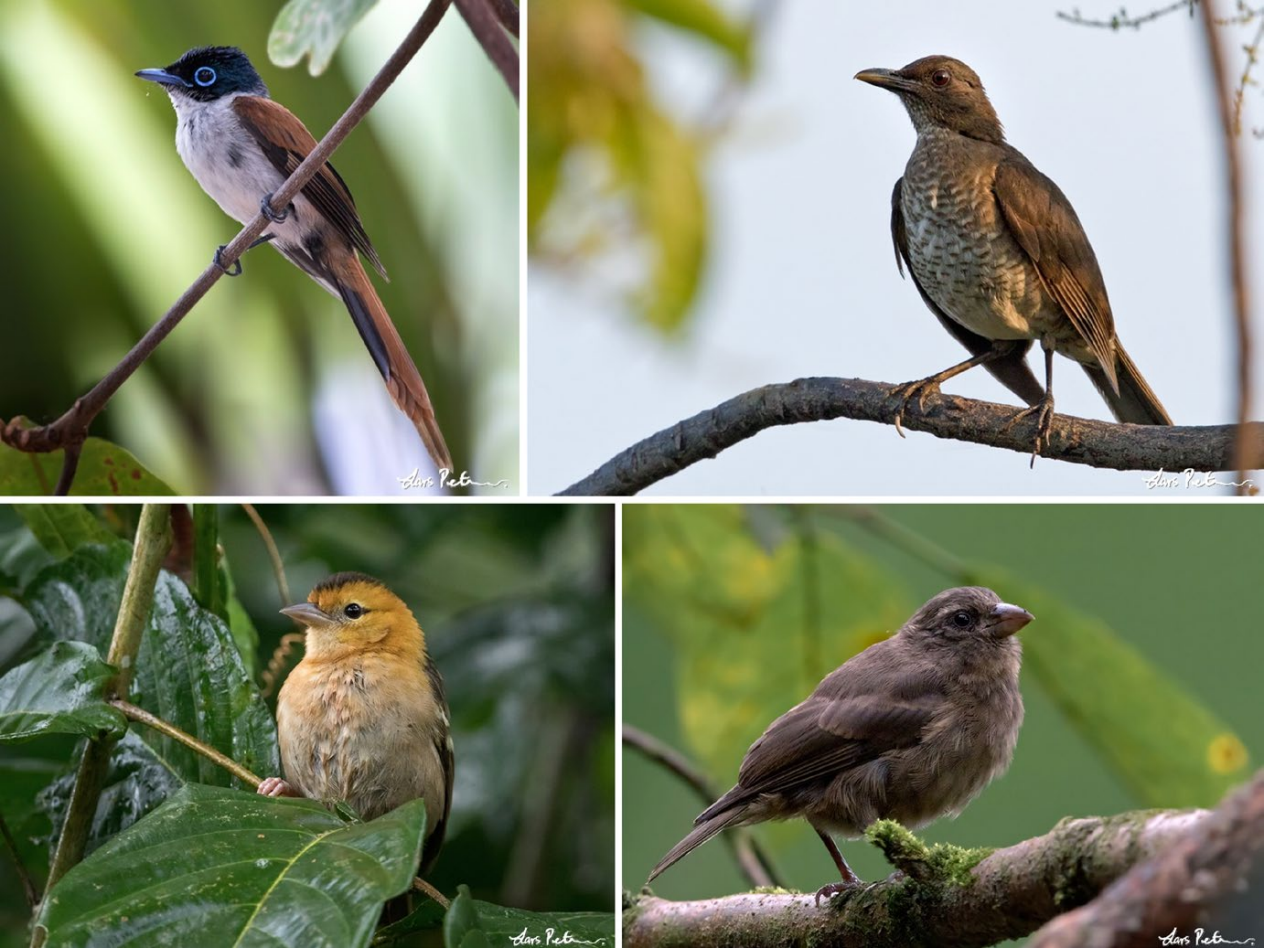
Bird species whose blood or fecal samples were used to assess different primer sets for eukaryotic endoparasite metabarcoding. Top left: São Tomé paradise‐flycatcher (*Terpsiphone atrochalybeia*); top right: São Tomé thrush (*Turdus olivaceofuscus*); bottom left: São Tomé weaver (*Ploceus sanctithomae*); bottom right: Principe seedeater (*Crithagra rufobrunnea*). Original pictures by Lars Petersson, reproduced with permission

Bird samples were collected on São Tomé Island (Gulf of Guinea, Africa). Species (illustrated on Figure 1), tissue, and collection year for each sample are summarized in Table 1. Samples were preserved in ethanol, and DNA was extracted using the PureLink Genomic DNA Mini Kit (Invitrogen) for blood samples, and the Stool DNA Isolation Kit (Norgen) for fecal and nematode samples, following manufacturer's instructions.

**TABLE 1:** List of bird samples used to test primers for parasite metabarcoding

Sample name	Sample type	Species	Collection year
B_Cri_1	Blood	Principe seedeater (*Crithagra rufobrunnea* )	2014
B_Cri_2	Blood	Principe seedeater (*Crithagra rufobrunnea*)	2014
B_Plo_1	Blood	São Tomé weaver (*Ploceus sanctithomae*)	2019
B_Plo_2	Blood	São Tomé weaver (*Ploceus sanctithomae*)	2019
B_Ter_1	Blood	São Tomé paradise‐flycatcher (*Terpsiphone atrochalybeia*)	2019
B_Ter_2	Blood	São Tomé paradise‐flycatcher (*Terpsiphone atrochalybeia*)	2019
B_Tur_1	Blood	São Tomé thrush (*Turdus olivaceofuscus*)	2019
B_Tur_2	Blood	São Tomé thrush (*Turdus olivaceofuscus*)	2019
F_Tur_1	Feces	São Tomé thrush (*Turdus olivaceofuscus*)	2019
F_Plo_1	Feces	São Tomé weaver (*Ploceus sanctithomae*)	2019
F_Tur_2	Feces	São Tomé thrush (*Turdus olivaceofuscus*)	2019
F_Plo_2	Feces	São Tomé weaver (*Ploceus sanctithomae*)	2019
F_Ter_1	Feces	São Tomé paradise‐flycatcher (*Terpsiphone atrochalybeia*)	2019
F_Ter_2	Feces	São Tomé paradise‐flycatcher (*Terpsiphone atrochalybeia*)	2019
1in100	Mix	Newton's sunbird (*Anabathmis newtonii*) and Spirurida nematode^a^	Bird: 2017 Nematode: 2014
1in1000	Mix	Newton's sunbird (*Anabathmis newtonii*) and Spirurida nematode^a^	Bird: 2017 Nematode: 2014

^a^ Isolated from a *Gallotia stehlini* lizard.

The research complied with all relevant national and international legal requirements, and the protocol and procedures were ethically reviewed and approved by the CIBIO's research center focal point of RedeORBEA (Network of the Bodies Responsible for Animal Welfare). In accordance with the legal determinations, CIBIO complies with all the conditions spelled out in the Convention on Biological Diversity for the use of genetic resources, any associated Traditional Knowledge and the sharing of resulting benefits between the parties concerned.

### 2.2 Primers

We tested five different primer sets deemed promising for bird parasite metabarcoding (Table 2). The five primer sets were applied to the same above set of 16 samples.

**TABLE 2:** Primer sets tested for their efficacy in sampling avian endoparasites

Set name	Target taxa	Target gene	PCR type	Primer names	5’ to 3’ primer sequence, including any 5’ overhang or 3’ modification		Modal amplicon length	References
*xca*	Eukaryotes (broad)	18S, V7‐V8 region	Single round	MiniB18S_81F MiniB18S_81R	foh‐GGCCGTTCTTAGTTGGTGGA roh‐CCCGGACATCTAAGGGCATC		180 bp	Cabodevilla et al. (2020)
*nes*	Single‐celled eukaryotes	18S, V4 region	Nested	18S‐EUK581‐F 18S‐EUK1134‐R	GTGCCAGCAGCCGCG TTTAAGTTTCAGCCTTGCG		~560 bp	Del Campo et al. (2019)
				E572F E1009R	foh‐CYGCGGTAATTCCAGCTC roh‐CRAAGAYGATYAGATACCRT		420 bp	
*dc2*	Single‐celled eukaryotes	18S, V4 region	Nested	574*f UNonMet_DB	foh‐CGGTAAYTCCAGCTCYV roh‐CTTTAARTTTCASYCTTGCG		398 bp	Bass & del Campo (2020)
*bl3*	Eukaryotes (broad)	28S, D4‐D5 region	Single round	RM2F RM3R	foh‐AGGGGCGAAAGACYAATCGAA roh‐CRCCAGTTCTGCTTACCAAAA		241 bp	Kounosu et al. (2019)
				BL3	TTACCAAAAGTGGCCCACTGAGCA#			
*bl4*	Eukaryotes (broad)	28S, D4‐D5 region	Single round	RM2F RM3R	foh‐AGGGGCGAAAGACYAATCGAA roh‐CRCCAGTTCTGCTTACCAAAA		241 bp	Kounosu et al. (2019)
				BL4	CAAAAGTGGCCCACTGAGCACTCGC#			

Note

The PCR primers had 5′ overhangs to allow subsequent library preparation for Illumina sequencing. The 5′ overhang on the forward primer, coded “foh” in the table, was TCGTCGGCAGCGTCAGATGTGTATAAGAGACAG (all sequences in this report are given from 5′ to 3′) and the 5′ overhang on the reverse primer, coded “roh,” was GTCTCGTGGGCTCGGAGATGTGTATAAGAGACAG. # indicates a C3 spacer at the 3′ end of the blocking primers.

Primer set 1, here coded “xca,” was developed for the simultaneous analysis of the intestinal parasites and diet from the feces of farmland birds through environmental DNA metabarcoding (Cabodevilla et al., 2020). It is a generalist eukaryotic primer pair targeting a short region of the 18S gene (~180 bp amplicons; Figure S1). It is capable of amplifying a wide range of eukaryotic organisms from unicellulars to plants, fungi, and metazoans, while excluding bacteria and Archaea. It has been shown to amplify various potentially parasitic groups such as apicomplexans, nematodes, and platyhelminthes.

Primer set 2, coded “nes,” was developed to study animal‐associated micro‐eukaryotic communities (del Campo et al., 2019). This primer set is designed to amplify unicellular eukaryotes only, thus excluding host DNA, but also potential metazoan parasites, such as helminths. It entails a nested PCR with a first round using a set of unicellular‐specific primers to exclude metazoans, followed by a second round using the primers of Comeau et al. (2011), also developed to study micro‐eukaryotic communities. In silico analyses suggested that this set could amplify potentially parasitic groups such as Alveolata or Heterokonta; empirical analysis of human fecal samples yielded less than 10% human reads.

Primer set 3, coded “dc2,” was also developed to retrieve micro‐eukaryotes (and exclude metazoans) from animal‐ and plant‐associated microbiomes, using a single PCR round (Bass & del Campo, 2020). In silico analyses also suggested a good sequence match with potentially parasitic groups such as Alveolata and Heterokonta.

Whereas primer sets 1–3 above targeted the 18S gene, primer sets 4 and 5 both targeted the 28S gene. Both used the same PCR primers RM2F and RM3R described in Kounosu et al. (2019) and deemed to amplify efficiently both Apicomplexa and helminths, while offering a reasonable amplicon length (~240 bp) and excluding bacterial sequences. These primers were shown to effectively amplify nematodes, platyhelminthes, and coccidia. In an attempt to improve their specificity by reducing amplification of host DNA, we designed two different blocking primers (Figure S2) to prevent annealing of the reverse PCR primer on bird sequences, resulting in primer sets 4 (“bl3”) and 5, (“bl4”) respectively. Blocking primers are oligonucleotides that are modified at their 3′ end so that they cannot initiate elongation during PCR; they are designed to match the sequence of unwanted templates, but not that of target templates. They shall therefore selectively block amplification of nontarget sequences during PCR, either by competition with the forward or reverse PCR primer during annealing, or by preventing complete elongation along the nontarget DNA strands (Vestheim et al., 2011). Further primer description is provided in Table 2 and Table S1.

### 2.3 Library preparation and next‐generation sequencing

For primer sets *xca*, *nes* (first round), and *dc2*, PCR were carried out in reactions containing 5 µl 2× Qiagen Multiplex PCR Master Mix with a final concentration of 0.4 µM of each forward and reverse primer. For the *bl3* and *bl4* primer sets, reactions were carried out as above, except that the respective blocking primers were also included to a final concentration of 12 µM. Either 1 or 2 µl DNA extracts were used as template, respectively, for blood and fecal samples (which usually contain less DNA than blood samples), and water was added to a final reaction volume of 10 µl. The methods described here are intended to be applied to samples whose status for any given parasite clade has not been previously assessed. Therefore, template DNA amount was not adjusted according to any previous information on, for example, parasitemia; back titrations indicated that the total template DNA amount ranged between 8 and 78 ng per reaction. For the *nes* primer set (second round), PCR were carried out in reactions containing 5 µl 2× Kapa HiFi HotStart ReadyMix Mix (Roche) with a final concentration of 0.5 µM of each forward and reverse primer. Two microliters of round one PCR products were used as template, and water was added to a final reaction volume of 10 µl. Cycling conditions varied between primers and are detailed in Table S1.

PCR primers included 5′ overhangs (Table 2) to allow subsequent library preparation for Illumina sequencing. Following the first PCR, amplicons were directly indexed (without intermediate cleaning step) with i5 and i7 indexes. Indexing PCR were carried out in 10 µl reactions containing 5 µl 2× Kapa HiFi HotStart ReadyMix, 0.5 µM each indexing primer, 2 µl PCR product as template, and 2 µl water. Primers were P5: 5′‐AATGATACGGCGACCACCGAGATCTACACxxxxxxxTCGTCGGCAGCGTC‐3′ and P7: 5′‐CAAGCAGAAGACGGCATACGAGATxxxxxxxGTCTCGTGGGCTCGG‐3′ as in da Silva et al. (2019), where xxxxxxx indicates the seven‐base sample‐specific indexes. Cycling consisted of a 3‐min incubation at 95℃, followed by nine cycles each consisting of 30 s at 95℃, 30 s at 55℃, and 30 s at 72℃, followed by a final elongation step of 5 min at 72℃. Indexed products were then cleaned using the AMPure XP beads (Beckman Coulter) and quantified using the Epoch Microplate pectrophotometer. Samples were pooled by barcode to an equal concentration of 0.25 nM of each sample in the pool, and product sizes were ascertained using the TapeStation (Agilent). Pool concentrations were quantified more precisely for Illumina sequencing using qPCR. Sequencing was carried out on a MiSeq (M01998) machine using a Nano v2 500‐cycle kit, with a PhiX spike‐in of 15% by molarity.

### 2.4 Data analysis

Sequences were demultiplexed using *bcl2fastq,* and adapters were trimmed with the Illumina software. For each primer set, paired‐end reads were merged accepting a 10% mismatch rate using *vsearch*. Only merged reads of a length comprised between 50 and 500 bp and containing both forward and reverse PCR primers (allowing a 10% mismatch) were kept, and primers were then removed from these sequences using *cutadapt*. Reads containing ambiguous nucleotides were also removed. Reads were clustered using *swarm* following a two‐step procedure, first with aggregation distance = 1 and then with aggregation distance = 3. *vsearch* was then used to identify and remove clusters whose seed sequence was deemed chimeric. The remaining clusters were affiliated using BLAST and the Silva 138 or 138.1 database (https://www.arb‐silva.de/), resulting in an affiliated OTU (Operational Taxonomic Unit) table for each primer set. These analyses were carried out using the FROGS suite in Galaxy (Escudié et al., 2018). Subsequent analyses on the affiliated OTU tables were carried out using *R*. The OTU table taxonomy information was manually curated, and the datasets were trimmed to the twenty most abundant OTUs for each primer set × sample type (blood, fecal, or mix) combination for analyses.

## 3 RESULTS

### 3.1 Sequencing and filtering metrics

We carried out one sequencing run that included all primer sets. A total of 2,157,180 reads were obtained, of which 2,038,148 (94.48%) passed the Illumina “chastity filter”. Error rate was 1.08% and, average proportion of ≥Q30 bases was 84.90%. These sequencing reads, in *fastq* format, are available from the Bourret et al. (2021) dataset.

Between 114,461 and 179,008 read pairs were obtained for each primer set (for the 16 samples combined). These were kept if they could be paired‐end assembled, contained both the forward and reverse primer sequences, were 50–500 nucleotides long, had no ambiguous base calls, and were deemed nonchimeric. The proportion of reads passing these filters varied between 66% and 92% for four of the primer sets, while it was 37% for primer set *dc2*. This was due to a substantial number of reads being eliminated because they were less than 50 nucleotides long. The remaining sequence clusters (hereafter referred to as Operational Taxonomic Units, OTU) were subsequently affiliated using the SILVA database (Quast et al., 2013). While 97%–99% of reads were affiliated for four of the primer sets, only 1% of reads were affiliated in the case of *dc2*; this marker was subsequently abandoned. Table 3 provides a summary of the number of reads kept and affiliated for each primer set.

**TABLE 3:** Performance of the various primer sets tested for the detection of avian eukaryotic endoparasites

Primer set	Initial read pair number	Read pairs kept (average per sample)	Proportion affiliated	No. different parasite families in blood samples	No. different parasite families in fecal samples
*xca*	172,319	158,959 (9,935)	99%	3	3
*nes*	179,008	119,171 (7,448)	97%	2	1
*dc2*	114,461	42,733 (2,670)	1%	n.a	n.a
*bl3*	119,190	100,086 (6,255)	99%	4	2
*bl4*	156,153	135,632 (8,477)	99%	4	3

### 3.2 Blood samples

**FIGURE 2:**
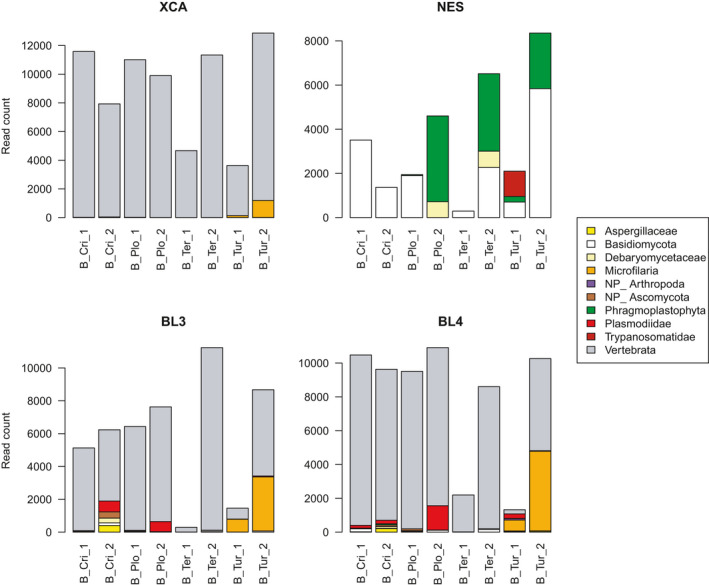
Read counts across eight blood DNA samples from four bird species for the four metabarcoding primer sets *xca*, *nes*, *bl3,* and *bl4*, showing the most prevalent taxonomic groups detected. NP, nonparasitic. Cri: *Crithagra rufobrunnea*; Plo: *Ploceus sanctithomae*; Ter: *Terpsiphone atrochalybeia*; Tur: *Turdus olivaceofuscus*

Figure 2 shows that most (84%–98% of total depending on the primer) sequences returned by primer sets *xca*, *bl3,* and *bl4* were from the avian hosts. This was not the case with the *nes* set, where host sequences were absent; this confirms the efficacy of this primer set at avoiding amplification of metazoan sequences. The most abundant clusters returned by *nes* were affiliated to *Malassezia* (Basidiomycota) which made up 55% of reads. *Malassezia* is a mostly commensal yeast genus, associated with the skin of warm‐blooded animals (Torres et al., 2020). Some *Malassezia* species have birds as their main host (Theelen et al., 2018), whereas others have been associated with systemic infection including fungemia in humans. To date, fungemia has not been reported in birds, however, and the *Malassezia* detected here could be contaminants from the skin of birds (or field workers), rather than bird blood parasites.

In terms of blood parasite detection, the *xca*, *bl3,* and *bl4* primers all detected substantial microfilaria DNA in both *Turdus* individuals (4,710 microfilaria reads were detected in individual Tur_2 by *bl4*; Figure 2). Consistent with its bias against metazoans, the *nes* primer set failed to detect any microfilaria nematodes. Of note, extremely low microfilaria read counts were also returned by *bl3* in Cri_2, Plo_1, and Ter_1 (one single read in each individual), and *bl4* in Plo_1 and Ter_1 (two reads each). Such low read counts are invisible in Figure 2. Detailed read counts are provided in Table S2.

Plasmodiidae, another major blood parasite group, was detected with substantial read numbers in three individuals using the *bl3* set and in four individuals using the *bl4* set (up to 1,434 reads in a single individual). Lower levels were also detected by *bl3* in Cri_1 and Tur_1 (30 and eight reads, respectively) and by *bl4* in Tur_2 (34 reads). A diversity of plasmodiid sequences was retrieved with *bl3* and *bl4*, consisting of five different clusters affiliated to either *Plasmodium* (four different clusters, of which three were hosted by a single *Ploceus* individual) or *Haemoproteus* (one cluster). Despite being unicellular, these malaria parasites were not detected with the *nes* primer set. On the other hand, the *nes* set was the only one to detect infection of the Tur_1 individual by *Trypanosoma*, with over a thousand reads.

Various primer sets also allowed detection of fungal families that include bird pathogens, namely Aspergillaceae or Debaryomycetaceae which, respectively, host the avian pathogens *Aspergillus fumigatus* (Beernaert et al., 2010) and *Candida* *albicans* (Jacobsen et al., 2008). Finally, a substantial number of plant DNA reads (up to 3,882 reads in one individual) occurred with the *nes* primers, which was not expected; most of these plant amplicons were less than 250 nt long, that is, substantially shorter than the main 420 nt *nes* peak (Figure S1).

### 3.3 Fecal samples

**FIGURE 3:**
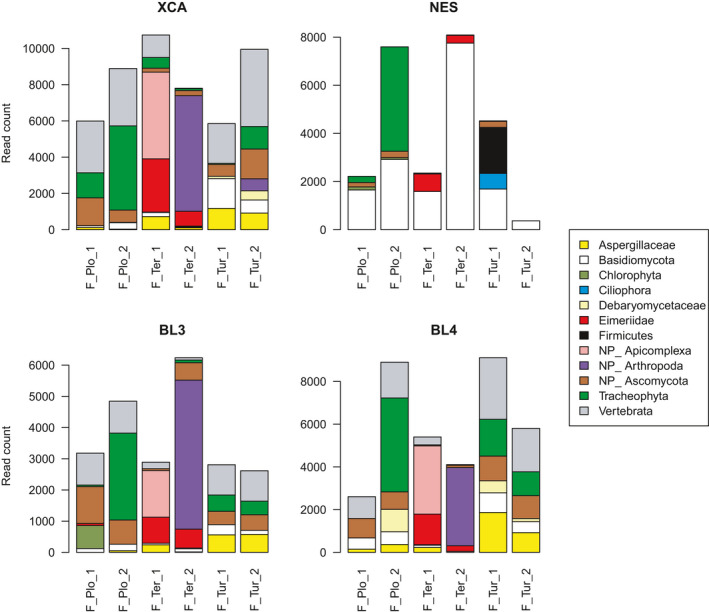
Read counts across six fecal DNA samples from three bird species for the four metabarcoding primer sets *xca*, *nes*, *bl3,* and *bl4*, showing the most prevalent taxonomic groups detected. NP, nonparasitic. Plo: *Ploceus sanctithomae*; Ter: *Terpsiphone atrochalybeia*; Tur: *Turdus olivaceofuscus*

Figure 3 shows the number of reads obtained for the most prevalent taxonomic groups detected in six bird fecal DNA samples. The diversity of taxa detected was generally higher than with blood samples and included parasite, host, and diet‐related items.

Parasitic coccidia such as *Eimeria* or *Isospora* (family Eimeriidae) were detected in the two *Terpsiphone* individuals with all four primer sets. Remarkably, all four sets consistently returned a higher Eimeriidae read count for individual Ter_1 than individual Ter_2. Besides these two individuals, the *bl3* set also detected a sizeable *Eimeria* population in Plo_1 (67 reads).

The three primer pairs *xca*, *bl3*, and *bl4* returned notable numbers of Aspergillaceae DNA reads, most consistently in both *Turdus* and one *Terpsiphone* individuals. Debaryomycetaceae were also recovered, notably in both *Turdus* individuals by both *xca* and *bl4*. All primers retrieved other Ascomycota (up to 1,641 reads in one individual with *xca*), belonging to a variety of taxa that were not bird parasites but rather plant pathogens such as *Cladosporium* or *Ramularia*. The *nes* primer set also returned large numbers of fungi reads from the phylum Basidiomycota. These reads were from taxa that are not known bird pathogens, including some *Malassezia* alongside taxa from the Auriscalpiaceae family—which interestingly are plant pathogens and wood saprotrophs (Nguyen et al., 2016), and therefore likely came from the diet.

The *nes* primer set identified a ciliate population (phylum Ciliophora) in one *Turdus* individual. These reads were affiliated to class Colpodea, a group of ciliates that are common in freshwater and soil habitats but generally not considered pathogenic. Other Ciliophora pathogenic in birds, such as *Balantidium* (class Litostomatea) (Marietto‐Gonçalves et al., 2008), were not detected. Of note, a substantial *Enterococcus* (bacterial) population (1,911 reads) was also detected with the *nes* primers in the same individual, whereas no bacteria were found with the other primers.

Diet or diet‐related items were abundantly detected with most primers. For instance, relatively abundant plant DNA was recovered from *Ploceus* and *Turdus* individuals with most primers, while it was much less abundant in the insectivorous *Terpsiphone* flycatchers. Individual Ter_2 had a large number of arthropod reads, consistent with its diet. Individual Ter_1 had a large number of reads allocated to noncoccidian apicomplexans with the three primer sets *xca*, *bl3,* and *bl4*. These reads were affiliated to order Arthrogregarida; this group (related to the Cryptogregarida that infect birds) include parasites of arthropods, which in turn are part of the flycatcher's diet.

### 3.4 Artificial DNA mixes

**FIGURE 4:**
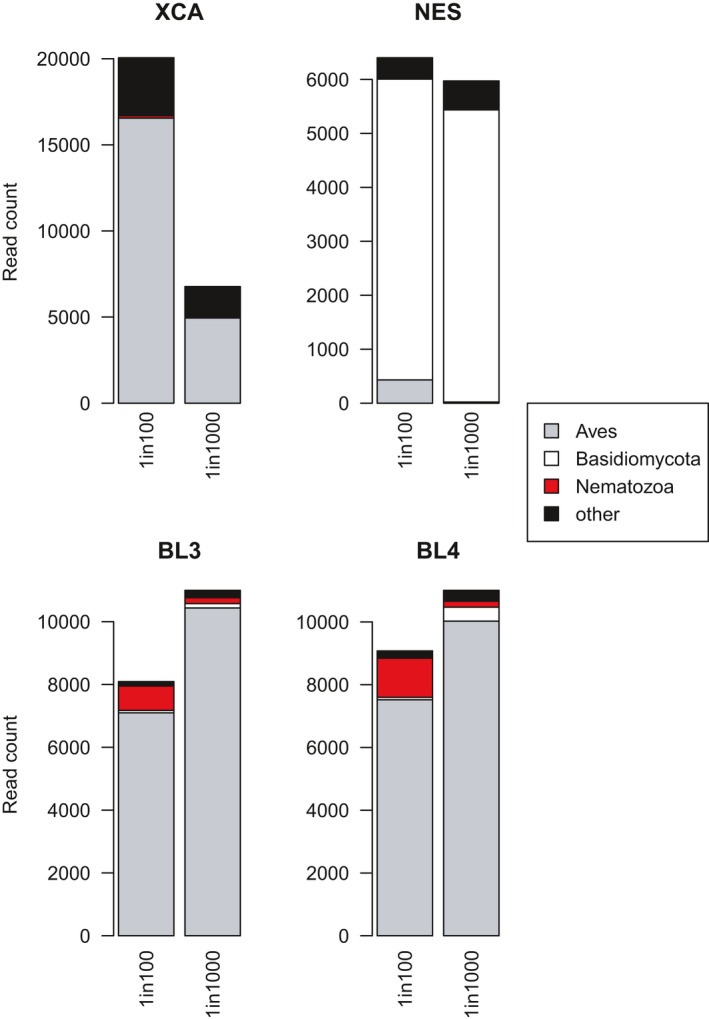
Read counts from two DNA mixes consisting of *Anabathmis* (bird) DNA (99% or 99.9%) spiked in with Spirurida nematode DNA (1% or 0.1%, respectively), retrieved by the four metabarcoding primer sets *xca*, *nes*, *bl3,* and *bl4*

We quantified the ability of the four primer sets to detect a known, low proportion (1% or 0.1%) of target (helminth) DNA in the presence of a high proportion of nontarget (bird) DNA. The best results were obtained with the *bl3* and the *bl4* primer sets since they returned the most nematode reads for both mixes (Figure 4). As expected, the *nes* primers did not return any nematode read from either mix, whereas *xca* maintained the nematode reads close to their original proportions (0.7% in the 1% mix and 0.28% in the 0.1% mix, Table 4). On the other hand, the *bl3* set increased the proportion of nematode reads to 9.6% in the 1% mix, and 1.69% in the 0.1% mix (*i.e*., roughly a 17‐fold increase). The *bl4* set also increased the nematode DNA proportions, to 13.7% in the 1% mix, and to 1.71% in the 0.1% mix (also a 17‐fold increase).

**TABLE 4:** Nematode read count and fold increase obtained in two samples consisting of bird DNA spiked in with either 1% or 0.1% nematode DNA, using the four metabarcoding primer sets *xca*, *nes*, *bl3,* and *bl4*

Set name	1% mix		0.1% mix	
	Nematode reads	Fold increase	Nematode reads	Fold increase
*xca*	143	0.7	19	2.8
*nes*	0	n.a	0	n.a
*bl3*	780	9.6	186	16.9
*bl4*	1,247	13.7	189	17.1

Reads from taxa other than bird or nematode were retrieved with all four primer sets, although their identity and proportions were variable. As expected, the *nes* primers yielded very few host (bird) reads, and instead returned mostly *Malassezia*. The *bl3* and *bl4* primers also yielded some *Malassezia*, while *nes*, *bl3,* and *bl4* detected tropical lichens (Ascomycota), and the *xca* set returned some unexpected DNA reads from a variety of nonbird vertebrate groups.

## 4 DISCUSSION

We tested five different primer sets for avian eukaryotic endoparasite metabarcoding in order to shed light on the usefulness of the data that can be obtained from the different sets and help researchers choose the most appropriate for their aims. For that purpose, eight blood samples, six fecal samples, and two controlled “bird+nematode” mixes were analyzed, and the outcome (in terms of parasite detection) was compared, revealing the pros and cons of each primer set.

Different criteria can be used to compare the parasite detection capacity of different primers for a given sample type (e.g., blood or feces). Those include the following:

- the number of different parasite clades detected,
- the total number of parasite reads across all samples taken together,
- the overall proportion of parasite reads across all samples taken together,
- the average of parasite read proportions across individual samples,
- the number of positive individuals.

To apply those criteria, the notions of “parasite clade” and “parasite reads” need to be defined, in the context of a metabarcoding analysis that is contingent upon the phylogenetic resolution of the markers and the completeness of the reference database. In this study, “parasite clade” is meant as any taxonomic family which includes known avian eukaryotic endoparasites; and a “parasite read” is a sequencing read affiliated to a parasite clade.

Criterion (i) (the number of different parasite clades) clearly seems relevant to the description of the global biodiversity of parasite communities and could arguably be used first; Table 3 shows how the primers tested here fared according to this criterion. Criteria (ii) and (iii) (the overall number or proportion of parasite reads, respectively) give an overview of the capacity of a primer set to yield workable numbers of parasite reads. We think that, provided the same sequencing effort is applied to the different test primers and samples, the total number of reads is preferable since it will also account for the efficacy of the whole process (from PCR to sequencing), directly answering the question of how many parasite reads can be obtained. The proportion‐based criteria (iii) and (iv) may be used if the sequencing effort is different from primer to primer, with criteria (iv) or (v) being preferable if the sequencing depth is substantially different between samples. For criterion (v) (the number of positive individuals), positivity needs to be defined in terms of the minimum number (or proportion) of parasite reads granting a sample the “positive” status. While in theory an individual with a single parasite read can be counted as positive, a higher threshold can be used in an effort to increase specificity. While many studies simply disregard single reads, defining such a threshold is in fact not trivial (Alberdi et al., 2018; Deagle et al., 2019), and the impact of threshold choice on real and artefactual biodiversity detection should be assessed using controlled DNA mixes.

Our results suggest that primer sets *bl3* or *bl4* are good options to study highly diverse blood parasites (Figure 2 and Table 3). For fecal samples, both *xca* and *bl4* returned three parasite clades (including fungi); however, the mix experiment showed that the *bl4* set has a stronger bias towards nematodes compared to the host, so we would recommend using this set. It is apparent from this study that no single primer set was infallible however. For instance, both *bl3* and *bl4* missed the *Trypanosoma* infection detected by *nes*, a marker which may therefore prove interesting if researchers wish to focus on some specific clades (having also shown a capacity to detect Ciliophora, a phylum which includes the digestive parasite *Balantidium*). Depending on the researchers' needs, other molecular parasite detection strategies can also be considered. For instance, a nested PCR combined with restriction‐based parasite DNA enrichment has been applied to human blood samples, detecting *Plasmodium*, *Trypanosoma*, and filarial nematodes (Flaherty et al., 2021). Likewise, the penguin diet primers of Jarman et al. (2013) have good potential to detect fecal parasite populations. Another possible approach is to carry out multiple targeted PCR to specifically detect various parasite groups, as done by Cannon et al. (2018).

Some fascinating co‐infection patterns were detected in the small sample set analyzed here. Figure 2 shows that one *Turdus olivaceofuscus* individual (Tur_1) was simultaneously infected with microfilaria (Animalia), Plasmodiidae (Chromista), and *Trypanosoma* (Protozoa) in the blood. Although the identification of these three extremely divergent parasitic taxa would be possible visually on a blood smear, applying this to many samples would be labor‐intensive. The metabarcoding approach also has the added benefit of providing genetic information on the parasites, which may, in some instances, shed some light on phylogenetic relationships among parasite lineages. In addition, both fungal families were present in the feces of that same bird (Figure 3), which therefore totaled co‐infections by five parasite clades from four different kingdoms. While *bl4* alone detected four of these five clades (80%), it took a combination of two markers (*bl4* and *nes*) to unravel the full breadth of this co‐infection. The *bl3* and *bl4* sets also revealed that one *Ploceus sanctithomae* individual (Plo_2) harbored three different *Plasmodium* clusters. Such co‐infections of individual birds with different *Plasmodium* lineages have been reported before using Sanger sequencing of the cytochrome *b*, based on the detection of double peaks (Reis et al., 2021; Rooyen et al., 2013). However, co‐infections may be missed using this method if a lineage is substantially more abundant or preferentially amplified compared to the others, resulting in a single visible peak (Bernotienė et al., 2016). The *bl3* and *bl4* set also revealed that the plasmodiid infecting both *Crithagra rufobrunnea* individuals was *Haemoproteus*, as opposed to *Plasmodium* in the other bird species. This is interesting to note since it shows that a single marker is capable of detecting both genera.

Clearly, a primary purpose of using these fragments is to diagnose infections by a broad range of parasite groups. It should be noted, also, that using primers able to detect simultaneously host, diet, and parasites of both the host and its diet (insects and plants), may allow a broader understanding of the ecosystem, by facilitating the integration of ecological and epidemiological data (Ezenwa, 2021). As an illustration of its use for ecological studies, we are currently applying the *bl4* set to a large number of samples from São Tomé to assess the impact of anthropogenic habitat modification (deforestation for oil palm monoculture) on bird parasite communities.

The use of these short fragments for parasite evolutionary analyses may also be possible, in instances where a given primer set enables detection of different OTU (or amplicon sequence variants) from the same parasite clade. For instance, the detection of several distinct *Plasmodium* OTU with the 28S primers may shed some light on their evolutionary relationships across bird samples. It should be noted on the other hand that individual apicomplexan genomes harbor several very different 18S gene copies, as investigated in *Plasmodium*, *Cryptosporidium*, and *Toxoplasma* (Nishimoto et al., 2008; Rooney, 2004; Stenger et al., 2015). As such, 18S sequence variation cannot be used to infer phylogenetic relationships between individuals in this group; 28S rDNA could be a preferable marker for that purpose, provided it does not display such intragenome heterogeneity (which to our knowledge has not been described, to date).

## 5 CONCLUSION

This side‐by‐side comparison of some promising primer sets for bird parasite metabarcoding showed that combining the 28S primers RM2F and RM3R (Kounosu et al., 2019) with a blocking primer offers an interesting option to describe major components of parasite biological diversity in the blood and feces, including helminths, apicomplexans, and fungi. This strategy also demonstrated potential to enrich an experimental mixture by returning more nematode reads than were initially present, and more than is returned by the *xca* set. This is promising in terms of the ability of these primers to detect co‐infections with helminths and malaria parasites, which will be interesting considering the known facilitation process by helminths in mammals including humans (Salazar‐Castañon et al., 2014). More generally, helminth infections are now being shown to have massive and far‐ranging implications, interacting with pathogens as diverse as coccidia (Clerc et al., 2019), highly pathogenic bacteria (Reynolds et al., 2017; Togarsimalemath et al., 2021), viruses (Hartmann et al., 2019), and even prions (Sánchez‐Quintero et al., 2019). The four‐kingdom co‐infection in one individual in this study, as well as the variety of plasmodiid strains detected in another sample, all show promise that metabarcoding has immense potential to reveal fascinating ecological interactions in bird parasite communities.

## CONFLICT OF INTEREST

The authors declare that there is no conflict of interest.

## AUTHOR CONTRIBUTIONS

**Vincent Bourret:** Conceptualization (equal); Data curation (equal); Formal analysis (equal); Investigation (equal); Methodology (equal); Writing‐original draft (equal); Writing‐review & editing (equal). **Rafael Gutiérrez López:** Investigation (equal); Writing‐review & editing (equal). **Martim Melo:** Investigation (equal); Writing‐review & editing (equal). **Claire Loiseau:** Conceptualization (equal); Funding acquisition (equal); Investigation (equal); Project administration (equal); Writing‐review & editing (equal).

## Supporting information

Supplementary Material

Table S2

## Data Availability

The sequencing data generated during this study and supporting the above discussion are publicly available through the Open Science Framework (OSF) at this URL: https://osf.io/9zxht/?view_only=c956dadb5f0a48d093bcf082ac5ab38b.
