# Supplementary material for: Metabarcoding options to study eukaryotic endoparasites of birds
Source: Ecol Evol. 2021 Jul 2;11(16):10821–33. doi: 10.1002/ece3.7748 (PMC8366860; doi:10.1002/ece3.7748)
Supplement: Supplementary file 1 — Supplementary Material [file ECE3-11-10821-s001.pdf]

Supplementary Materials

Metabarcoding options to study eukaryotic endoparasites in birds

Vincent Bourret, Rafael Gutiérez López, Martim Melo, Claire Loiseau

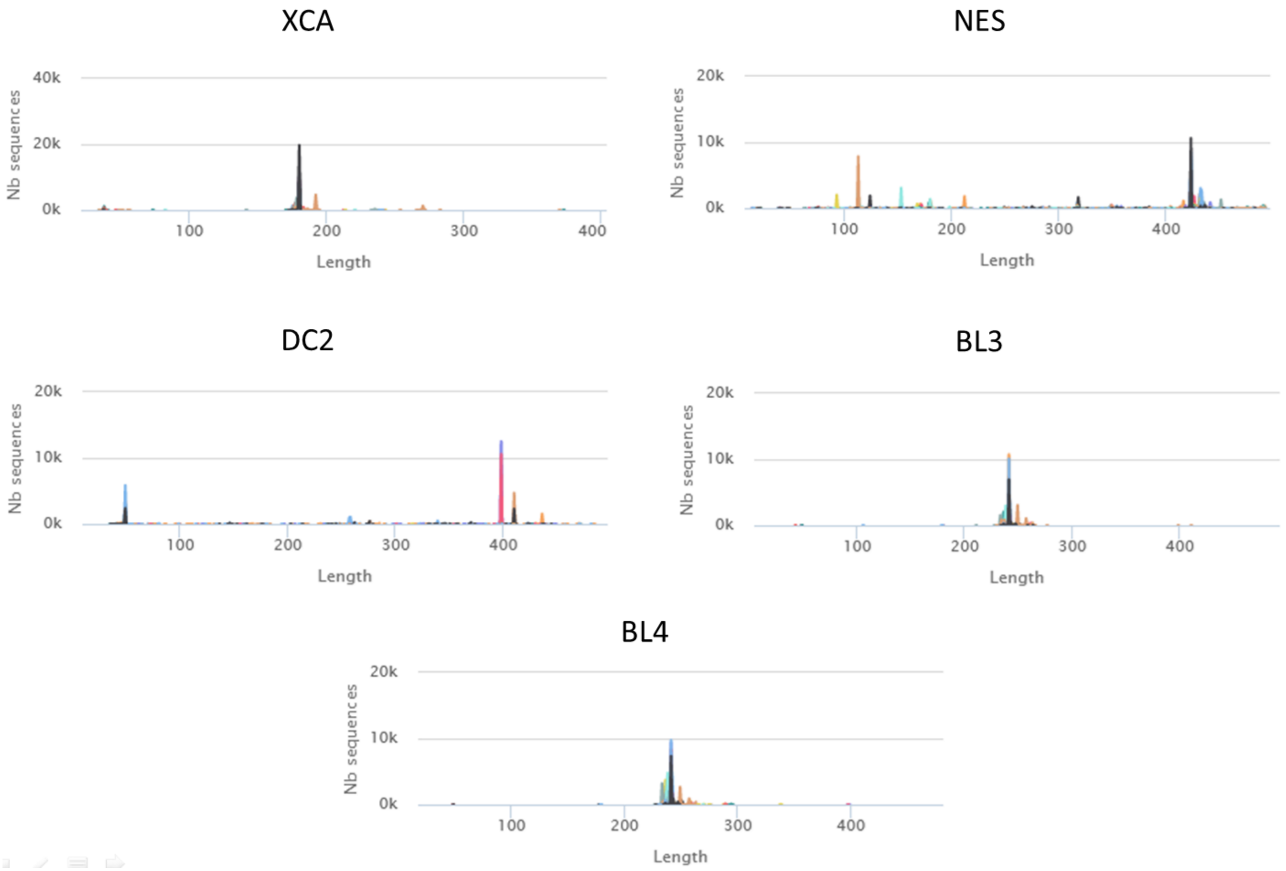

Figure S1. Distribution of amplicon lengths (before trimming and filtering) for the five candidate markers over 16 samples.

For the purpose of this comparison, we kept all reads 50-500 nt long for all markers, out of concern that potential parasite species could have undergone an alteration of the target region length, and to ensure that the same treatment was applied to the different primer sets being tested. For most markers, the amplicon lengths were normally distributed around a single mode (Figure S1). This was not the case with the '*nes*' primers however, which returned numerous reads shorter than 250 nt, while the main peak was at 420 nt. Most of these *nes* shorter reads in the blood samples were unexpectedly affiliated to plants (see Figure 1 in main text), mostly from widespread groups such as Leguminosae or Poaceae. These are groups whose range includes São Tomé and Príncipe (Figueiredo, Paiva, Stévart, Oliveira, & Smith, 2011) where the samples were collected, and these reads could therefore reflect environmental contamination. However, if the blood samples were indeed contaminated with plants, these should also be detected with other primer sets, which was not the case. Thus the origin of these plant reads in blood samples remains unclear, and should one wish to use the *nes* set, there is a case for considering only amplicons longer than ~250 nt (*i.e.* keeping only the main peak).

Figueiredo, E., Paiva, J., Stévart, T., Oliveira, F., & Smith, G. F. (2011). Annotated catalogue of the flowering plants of São Tomé and Príncipe. Bothalia, 41*(1), 41–82. doi:* [10.4102/abc.v41i1.34](10.4102/abc.v41i1.34)

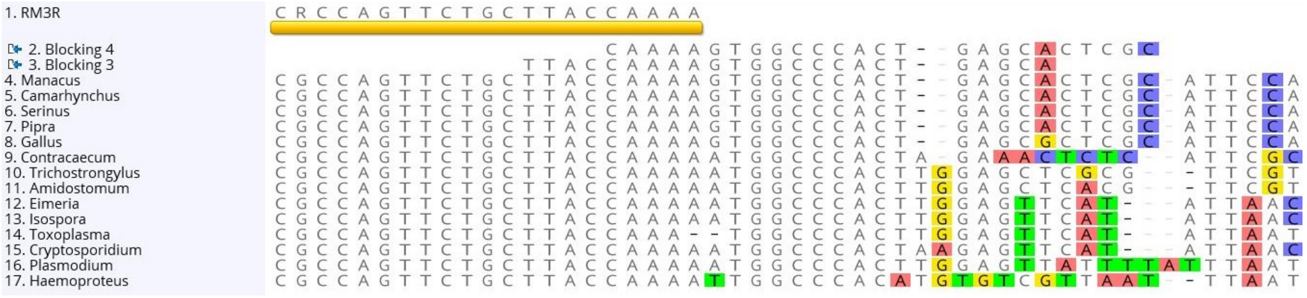

Figure S2. Alignment of the reverse primer region of the *bl3* and *bl4* amplicons. This alignment shows:

- The reverse PCR primer (named RM3R, sequence 1, underlined in yellow)
- The bl4 and bl3 blocking primers (sequences 2 and 3 respectively)
- Avian sequences (sequences 4 to 8)
- Parasite sequences (sequences 9 to 17).

Note the match between both blocking primer sequences and the avian sequences, and the multiple mismatches (including indels) between blocking primers and parasite sequences. The blocking primers (which cannot initiate elongation) are designed to anneal to host sequences only, thereby preventing annealing of the reverse PCR primer to these sequences and hence their amplification.

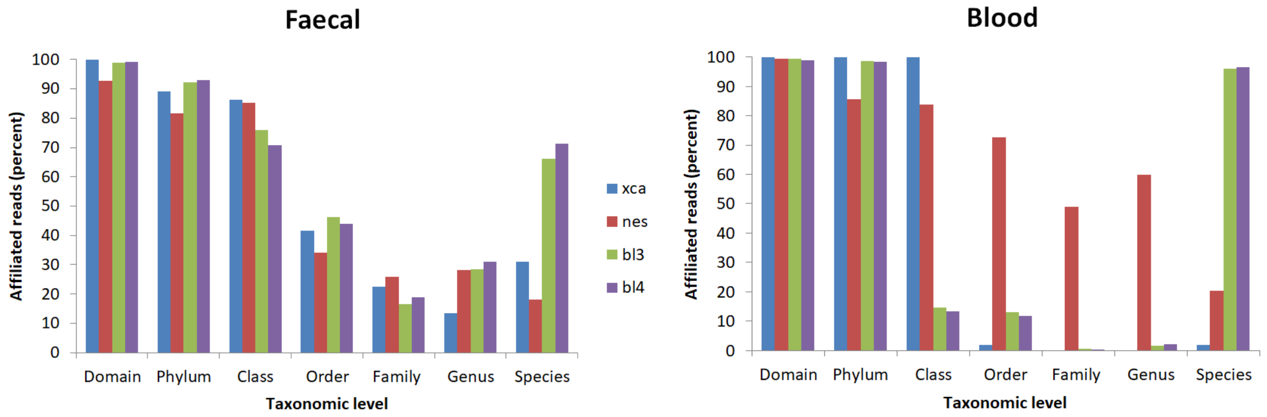

Figure S3. Percentage of the reads affiliated to various taxonomic levels for four metabarcoding primer sets.

Most faecal sample reads (70-86% depending on primer) were affiliated down to class level (*i.e.* had explicit, unambiguous taxonomical information down to class). There was a sharp decrease afterwards, with only 13-46% of the reads having taxonomical information at order or lower levels. This pattern was consistent for *xca* and *nes*, whereas 66% of reads for *bl3* and 71% of reads for *bl4* had taxonomical information at species level. Clearly when the species identity is known, higher taxonomic levels can generally be deduced so the SILVA 28S taxonomies need updating with more uniform taxonomic information (such as the one from EMBL-EBI/ENA databases). This update would have the potential to considerably reduce the manual curation work carried out between bioinformatics treatment and downstream statistical analyses.

Overall, the pattern was comparable with blood samples, except that the *nes* primers had a higher proportion of reads with order, family and genus identified, compared to the other primer sets.

Table S1. Information on primers tested in this study. PCR primers had 5' overhangs added to allow subsequent library preparation for Illumina sequencing. The 5' overhang on the forward primer, coded 'foh' in table S1, was TCGTCGGCAGCGTCAGATGTGTATAAGAGACAG (all sequences in this report are given from 5' to 3') and the 5' overhang on the reverse primer, coded 'roh', was GTCTCGTGGGCTCGGAGATGTGTATAAGAGACAG.

Set name	Primer names	5' to 3' primer sequence, including any 5' overhang or 3' modification	Target region	Modal amplicon length (bp)	Cycling conditions
xca	MiniB18S_81F<br>MiniB18S_81R	foh-GGCCGTTCTTAGTTGGTGGA<br>roh-CCCGGACATCTAAGGGCATC	18S,<br>V7-V8 region	180	95°C for 15 min, followed by 35 cycles of (94°C for 30 s, 51°C for 30 s, 72°C for 35 s) and a final extension step at 60°C for 5 min.
nes	18S-EUK581-F<br>18S-EUK1134-R	GTGCCAGCAGCCGCG<br>TTTAAGTTTCAGCCTTGCG	18S,<br>V4 region	~560	95°C for 15 min, followed by 16 cycles of (94°C for 30 s, 64°C for 30 s with a -0.5°C per cycle touchdown, 72°C for 45 s), 24 cycles of (94°C for 30 s, 56°C for 30 s, and 72 °C for 45 s) and a final extension step at 60°C for 5 min.
	E572F<br>E1009R	foh-CYGCGGTAATTCCAGCTC<br>roh-CRAAGAYGATYAGATACCRT		420	95°C for 3 min, followed by 22 cycles of (95°C for 30 s, 48°C for 30 s, 72°C for 40 s) and a final extension step at 72°C for 5 min.
dc2	574*f<br>UNonMet_DB	foh-CGGTAAYTCCAGCTCYV<br>roh-CTTTAARTTTCASYCTTGCG	18S,<br>V4 region	398	95°C for 15 min, followed by 8 cycles of (94°C for 30 s, 51°C for 30 s with a -0.5°C per cycle touchdown, 72°C for 30 s), 32 cycles of (94°C for 30 s, 47°C for 30 s, and 72 °C for 30 s) and a final extension step at 60°C for 5 min.[1]

[1]Since this primer pair yielded weak bands and failed to amplify some samples, we tried an alternative cycling profile recommended by the author (J. del Campo, pers. comm.), consisting of an initial denaturation step at 94°C for 10 min, followed by 40 cycles consisting of 94°C for 1 min, 62.5°C for 1 min, and 72°C for 1 min, followed by final extension at 72°C for 10 min. This failed to yield any stronger bands, and this alternative protocol was abandoned.

Table S1 ***continued***

Set name	Primer names	5' to 3' primer sequence, including any 5' overhang or 3' modification	Target region	Modal amplicon length (bp)	Cycling conditions
bl3	RM2F<br>RM3R	foh-AGGGGCGAAAGACYAATCGAA<br>roh-CRCCAGTTCTGCTTACCAAAA	28S,<br>D4-D5 region	241	95°C for 15 min, followed by 35 cycles of (94°C for 30 s, 68°C for 25 s, 60°C for 30 s, 72°C for 30 s) and a final extension step at 60°C for 5 min.
	BL3	TTACCAAAAGTGGCCCACTGAGCA-[C3 Spacer]			Blocking primer included in above PCR. Note the extra annealing step (68 °C for 25 s) added to favour blocking primer annealing before reverse PCR primer annealing.
bl4	RM2F<br>RM3R	foh-AGGGGCGAAAGACYAATCGAA<br>roh-CRCCAGTTCTGCTTACCAAAA	28S,<br>D4-D5 region	241	95°C for 15 min, followed by 35 cycles of (94°C for 30 s, 71°C for 25 s, 60°C for 30 s, 72°C for 30 s) and a final extension step at 60°C for 5 min.
	BL4	CAAAAGTGGCCCACTGAGCACTCGC-[C3 Spacer]			Blocking primer included in above PCR. Note the extra annealing step (71 °C for 25 s) added to favour blocking primer annealing before reverse PCR primer annealing.
